# Health of Louisville

**Published:** 1851-07

**Authors:** 


### HEALTH OF LOUISVILLE.

For two years in succession, namely in the summers of 1849 and 1850, cholera prevailed to some extent in this city. According to the course of the epidemic when it visited our country before, it ought not to appear here again in an epidemic form, and we are happy to state that, up to this time, although there have been a number of cases brought from the river, and a few scattering cases have originated in the city, no indication of an epidemic tendency has been observed. Dysentery has prevailed to a considerable extent, and diarrhœa has been general, but the latter, even when suffered to take its course, has seldom run into cholera. The indigenous cases of cholera, as a general rule, have not assumed the highly malignant character exhibited by the disorder when it is epidemic, but many of them have proved manageable at a stage in which all remedies were unavailing in former seasons. The health of the city is good for the summer season. Y.

